# MicroRNA-346 inhibits the growth of glioma by directly targeting NFIB

**DOI:** 10.1186/s12935-019-1017-5

**Published:** 2019-11-14

**Authors:** Yangyang Li, Jia Xu, Jiale Zhang, Jie Zhang, Jian Zhang, Xiaoming Lu

**Affiliations:** Department of Neurosurgery, The First Affiliated Hospital of Nanjing Medical University, Nanjing, 210029 Jiangsu China

**Keywords:** Glioma, miR-346, NFIB, Proliferation

## Abstract

**Background:**

Glioma is considered one of the most common tumors and has a poor prognosis. Recently, microRNAs (miRNAs) have been reported to be strongly linked to various human tumors including glioma. In this study, we investigated a new anticancer miRNA, miR-346, to determine the effects and mechanism of miR-346 and its downstream target gene NFIB on tumors.

**Methods:**

Lentivirus transfection, real-time PCR, western blotting, immunohistochemistry, cell proliferation assays, and mouse experiments were used to examine the relationship between miR-346 and its regulation of NFIB in glioma cells.

**Results:**

The expression of miR-346 was downregulated in glioma cells. Overexpression of miR-346 arrested the cell cycle of glioma cells and inhibited their proliferation in vitro and in vivo. NFIB was a direct target of miR-346, whose expression was reduced by the miRNA. Overexpression of NFIB reversed all tested functions of miR-346.

**Conclusion:**

miR-346 inhibited the growth of glioma cells by targeting NFIB and may be a new prognostic and diagnostic biomarker for glioma.

## Background

Glioma is one of the most common and malignant tumors in the adult brain and has a poor prognosis greatly reducing a patient’s lifespan [1, 2]. Glioma is usually classified into four grades. With the increase in grade, the malignant degree of glioma increases and the prognosis worsens [3]. Although significant progress has been made in the treatment of gliomas, such as surgical excision and chemotherapy, some patients still cannot receive effective treatment [4]. Therefore, new methods for diagnosis and treatment are necessary.

MicroRNAs (miRNAs) are a class of noncoding, single-stranded RNA molecules of approximately 22 nucleotides encoded by endogenous genes that participate in the regulation of posttranscriptional gene expression [5]. To date, 28,645 miRNA molecules have been found in plants, animals, and viruses [6]. Most miRNA genes exist in the genome as a single copy, multiple copies, or in a cluster [7]. miRNAs play important roles in various cellular functions, such as proliferation, invasion, and migration [8]. Furthermore, they have been reported to be closely associated with many kinds of tumors including glioma [9]. Strong evidence has shown that many miRNAs have positive or negative effects on glioma [10]. By comparing miRNA expression in different grades of glioma, studies have revealed potential miRNA functions. However, the mechanisms through which miRNAs affect glioma are still unknown [11].

NFIB, also known as human nuclear factor I, is an important regulator of lung and brain development [12]. Recent evidence has shown that NFIB gene expression is involved in the development of various types of tumors such as breast cancers, adenoid cystic carcinoma, and glioma [13]. However, the mechanism of NFIB in glioma remains unclear.

In this study, we focused on the effect of miR-346 on glioma cell growth and its tentative relationship with NFIB. We found that miR-346 inhibited the growth of human glioma cells. Moreover, overexpression of NFIB suppressed miR-346-regulated growth of human glioma cells. Our results suggest that miR-346 inhibits glioma cell growth and might provide a novel therapeutic method for glioma treatment.

## Methods

### Human tissue samples

miRNA expression data of 255 tissue samples were obtained from The Cancer Genome Atlas (TCGA) database (http://tcga-data.nci.nih.gov). Noncancerous brain tissues (n = 5 patients with brain trauma) and glioma specimens (low grade, n = 5; high grade, n = 10) were obtained from the First Affiliated Hospital of Nanjing Medical University, China. The use of human tissue samples was approved by the Research Ethics Committee of Nanjing Medical University (Nanjing, China). All procedures were performed under legal guidance. Patients had been treated for glioma for the first time and provided written informed consent. All tissue samples were frozen immediately after resection and stored in liquid nitrogen until analysis [14].

### Cell culture and antibodies

Human glioma cell lines U87, LN229, U251, A172, and U118 and 293T cells were obtained from the Chinese Academy of Sciences Cell Bank (Shanghai, China). Cells were cultured in Dulbecco’s modified Eagle’s medium supplemented with 10% fetal bovine serum, 100 U/mL penicillin, and 100 ng/mL streptomycin at 37 °C with 5% CO_2_. Normal human astrocytes were obtained from Lonza (Basel, Switzerland) and cultured in accordance with the supplier’s guidelines. Antibodies against NFIB, Cyclin E1, Cyclin D1, CDK4, and GAPDH were obtained from Cell Signaling Technology (MA, USA).

### Oligonucleotides, plasmid construction, and transfection

Lentiviruses carrying miR-346 or a miRNA negative control (miR-NC) were produced by RiboBio (Guangzhou, China). Small interfering (si) RNA targeting NFIB and control si-noncoding (siNC) oligonucleotides were purchased from GenePharma (Shanghai, China). Human NFIB cDNA was inserted into the pGL3 vector (GeneChem, Shanghai, China) to generate pGL3-NFIB. All oligonucleotides and plasmids were transfected using Lipofectamine 2000 Transfection Reagent (Invitrogen, CA, USA) according to the manufacturer’s instructions.

### Lentiviral packaging and establishment of stably transfected cell lines

A lentiviral packaging kit was purchased from GeneChem. A lentivirus carrying hsa-miR-346 or hsa-miR-negative control (miR-NC) was packaged in the human embryonic kidney cell line 293T. Virions were collected according to the manufacturer’s instructions. Stable cell lines were established by infecting U87 and U251 cells with lentiviruses, followed by puromycin selection.

### RNA isolation and quantitative reverse transcriptase-PCR

RNA was isolated from cell lines and fresh tissues using TRIzol reagent (Life Technologies, CA, USA) following the manufacturer’s protocol. Quantitative real-time PCR was conducted using the ABI StepOne Plus system (Applied Biosystems, CA, USA) with the Bulge-loop miRNA qRT-PCR Primer Kit (RiboBio, Guangzhou, China) to detect miR-346. Primers were purchased from RiboBio. Data were analyzed using the 2^−ΔΔCt^ method. U6 RNA was used as an endogenous control [15].

### Protein extraction and western blotting

Protein extraction and western blotting were performed as described previously [16]. Briefly, cells or tissues were lysed on ice for 30 min in radioimmunoprecipitation assay buffer [150 mM NaCl, 100 mM Tris, pH 8.0, 0.1% sodium dodecyl sulfate (SDS), 1% Triton X-100, 1% sodium deoxycholate, 5 mM EDTA, and 10 mM sodium fluoride]. The lysates were centrifuged at 12,000 rpm at 4 °C for 15 min, the supernatants were collected, and protein concentrations were determined using a bicinchoninic acid assay (KenGEN, Jiangsu, China). Equal amounts of protein were separated by 10% SDS-PAGE and then electrotransferred onto a polyvinylidene difluoride membrane (Thermo Fisher Scientific, MA, USA). Membranes were blocked with 5% dry nonfat milk for 2 h and then incubated with primary antibodies. An electrochemiluminescence detection system (Thermo Fisher Scientific) was used for signal detection.

### Cell proliferation and colony-forming assays

To assess cell proliferation, exponentially growing cells were seeded at 3000 cells per well and cultured in 96-well plates. Cell proliferation was measured using a Cell-Counting Kit 8 (CCK8; Dojindo Laboratories) according to the manufacturer’s instructions. The colony formation assay was performed as described previously. Briefly, 5 × 10^2^ cells were seeded in a 6-well plate. After 2 weeks, visible colonies were fixed with 100% methanol and then stained with 0.1% crystal violet for 30 min. Colony-forming efficiency was calculated as the number of colonies (diameter > 0.5 mm). To quantify DNA synthesis, a 5-ethynyl-2-deoxy-uridine (EDU) assay was performed using a Cell-Light EDU In Vitro Imaging Detection Kit (RiboBio, Guangzhou, China) according to the manufacturer’s instructions. Cells were imaged under a fluorescence microscope. All assays were repeated at least three times.

### Fluorescence in situ hybridization

The probes used for in situ hybridization to detect miR-346 were synthesized by GoodBio (Wuhan, China). Fresh tissues were fixed in 4% formaldehyde for 1 h and then dehydrated in 15% sucrose for 8 h. The tissues were fixed in 4% formaldehyde for 25 min, washed three times for 5 min each with phosphate-buffered saline (PBS; pH 7.4), digested using proteinase K at 37 °C for 5 min, and washed again three times for 5 min each using PBS. After eliminating autofluorescence and blocking endogenous biotin, the sections were hybridized with the probes overnight. The tissue sections were then washed with warm 2× saline sodium citrate (SSC) at 37 °C for 10 min, 1× SSC at 37 °C for 10 min, and then 0.5× SSC for 10 min. Finally, the tissue sections were counterstained with 4′,6-diamidino-2-phenylindole (Sigma) for 10 min and then examined under an LSM 700 Meta confocal microscope (Zeiss, Oberkochen, Germany).

### Flow cytometric analysis of cell cycle progression

Transfected cells were harvested, washed with PBS, fixed with 70% ice-cold ethanol, and stained using a Cell Cycle Staining Kit (Multi Sciences, Hangzhou, China) for 30 min in the dark before analysis by flow cytometry.

### Dual-luciferase reporter assay

Wild-type (WT) and mutated (Mut) putative miR-346 binding sites in the NFIB 3´-untranslated region (UTR) were amplified by PCR from human cDNA and inserted into the SacI and HindIII sites of the pmiRNA-Report firefly luciferase vector (GeneChem, Shanghai, China). U87 and U251 cells were seeded in 24-well plates and cotransfected with the WT or Mut reporter plasmids, a Renilla luciferase plasmid, and the miR-346 mimic or miR-NC. Luciferase activities were analyzed at 24 h after transfection using the Promega Dual-Luciferase Reporter Assay System (WI, USA).

### Nude mouse model of subcutaneous glioma

All animal experiments were approved by the Animal Management Rules of the Chinese Ministry of Health (document 55, 2001) and conducted in accordance with the approved guidelines and experimental protocols of Nanjing Medical University. Nude mice at 4 weeks of age were purchased from the Shanghai Experimental Animal Center of the Chinese Academy of Sciences and randomly divided into two groups. Mice were subcutaneously injected with 1 × 10^6^ U87 cells (pretreated with lentiviruses encoding miR-346 or miR-NC) bilaterally into axillary lymph nodes [17]. The mice were sacrificed at the end of the experiment, and their tumors were extracted, fixed in 10% formalin and then embedded in paraffin for hematoxylin and eosin staining and immunohistochemical analysis.

### Immunohistochemistry (IHC)

IHC detection was performed using antibodies against NFIB (Abcam) and Ki-67 (Cell Signaling Technology) in subcutaneous tumor tissues from nude mice as described previously [18].

### Statistical analysis

All experiments were performed three times. Values are presented as the mean ± standard deviation. Statistical analyses were performed using Student’s *t* test to evaluate significant differences between groups. Correlations between miR-346 expression and NFIB levels were assessed by GraphPad 5.0 software. P < 0.05 indicated a significant difference.

## Results

### miR-346 is downregulated in human glioma tissues and cell lines

**Fig. 1:**
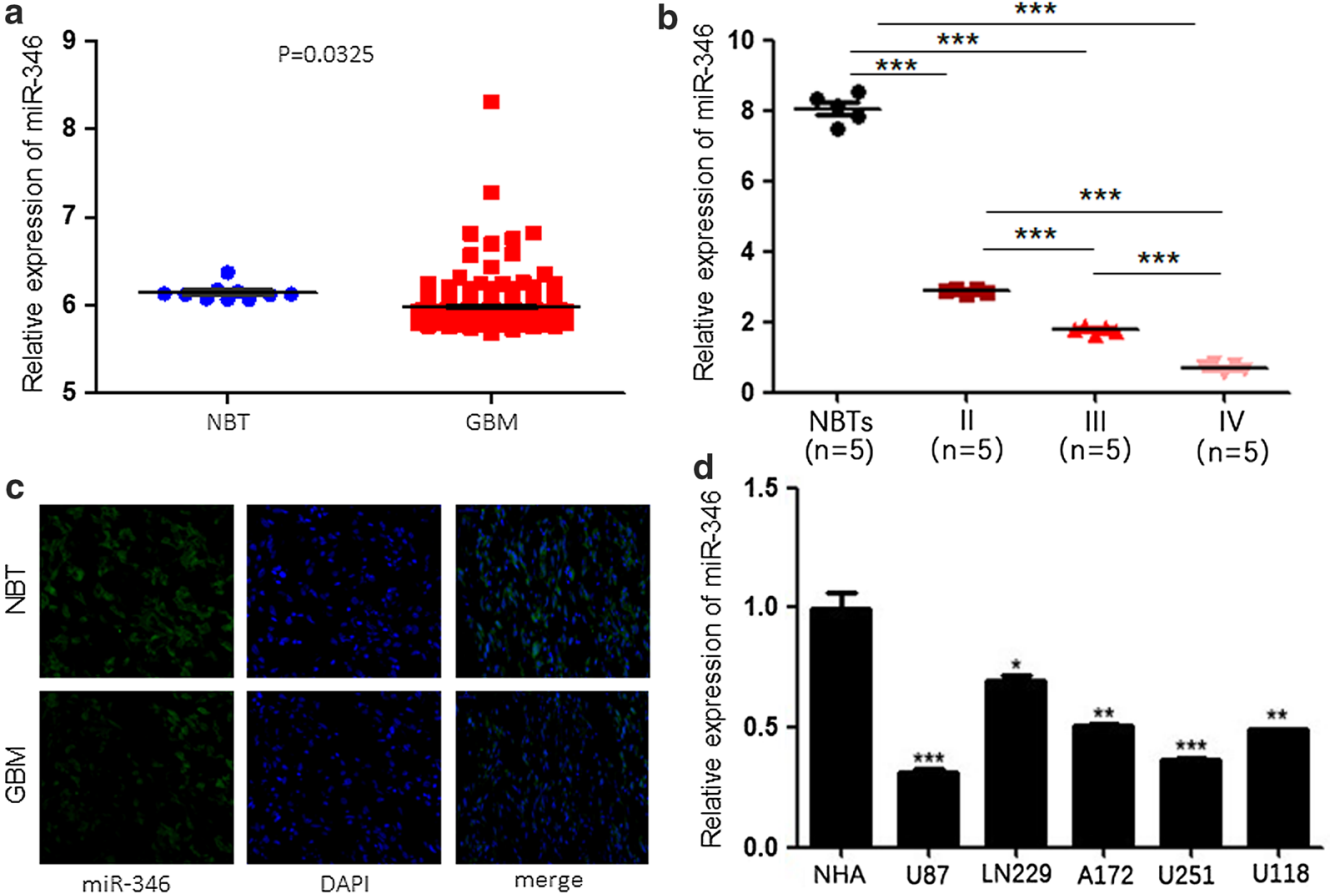
Downregulation of miR-346 in glioma tissues and cell lines. **a** qRT-PCR analysis of miR-346 expression in normal brain tissues (NBTs, n = 10) and glioma tissues (n = 255). **b** qRT-PCR analysis of miR-346 expression in NBTs (n = 5) and glioma specimens (n = 15) divided according to WHO pathological classification criteria into grade II (n = 5), grade III (n = 5), and grade IV (n = 5). **c** FISH analysis of miR-346 expression in glioma specimens. **d** qRT-PCR analysis of miR-346 expression in normal human astrocytes (NHAs) and five glioma cell lines (U87, LN229, A172, U251, U118). *P < 0.05, **P < 0.01, ***P < 0.001

To investigate miR-346 expression, we analyzed 255 glioma tissues based on The Cancer Genome Atlas (TCGA) data. miR-346 was significantly downregulated in GBM compared with noncancerous brain tissues (Fig. 1a). Moreover, we found that miR-346 expression was lower as the tumor grade increased (Fig. 1b). Next, we used GBM tissue and noncancerous brain tissue for FISH to confirm our TCGA data analysis (Fig. 1c). In addition, we assessed miR-346 expression in the human glioma cell lines U87, LN229, A172, U251, and U118. The results showed that miR-346 was decreased in all cell lines, especially U87 and U251 (Fig. 1d). These results suggested that miR-346 was downregulated in human glioma tissues and cell lines, which was linked to tumor grade.

### miR-346 overexpression suppresses cell proliferation

**Fig. 2:**
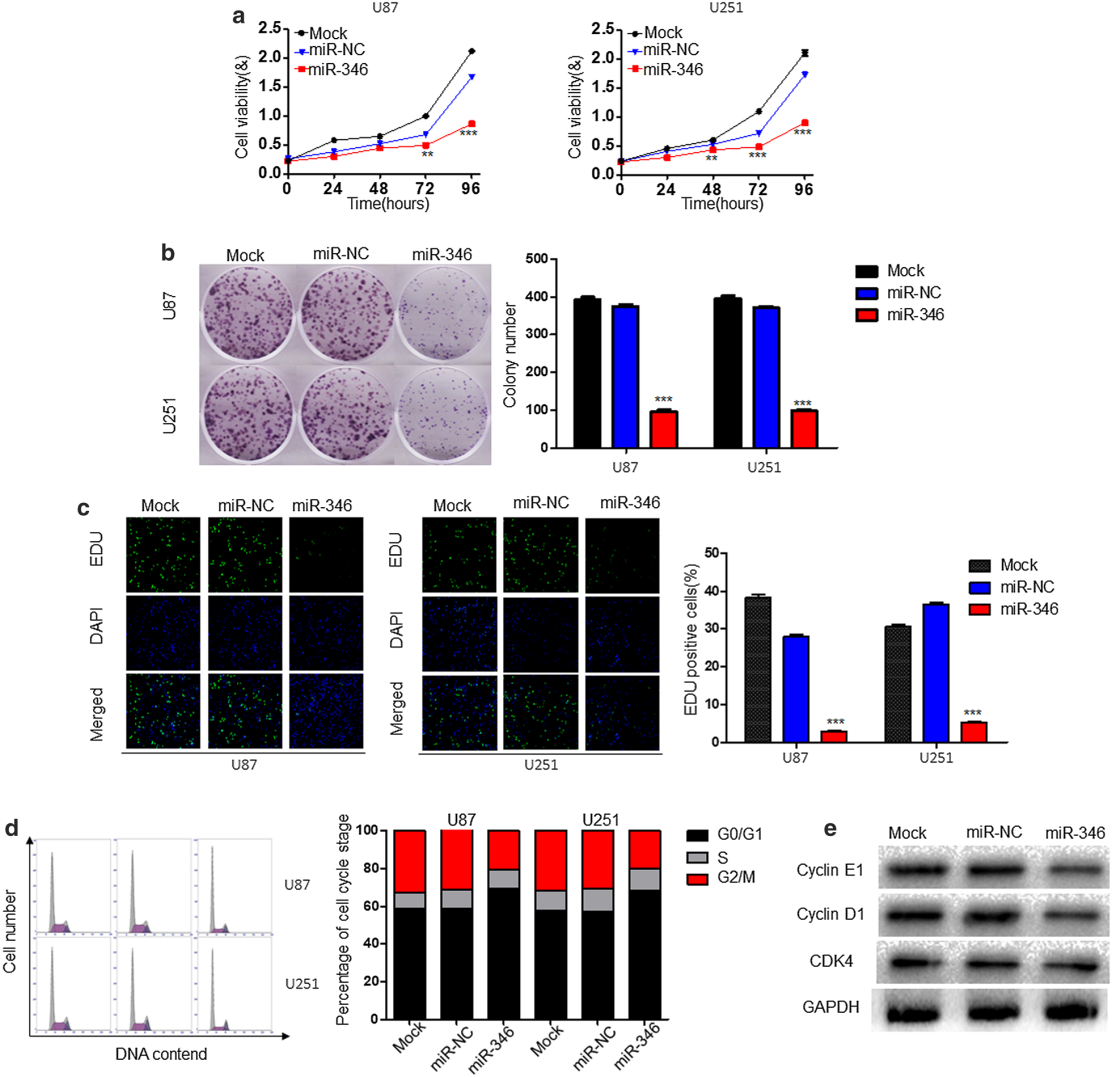
miR-346 overexpression induces cell cycle arrest and inhibits glioma cell growth in vitro. **a** CCK-8 assay of proliferation of U87 and U251 glioma cell lines transfected with miR-NC or miR-346. **b** Colony-forming assays of U87 and U251 cells transfected with miR-NC or miR-346. **c** Representative single or merged images of DAPI- and EDU-stained U87 and U251 cells transfected with miR-NC or miR-346. **d** Flow cytometric analysis of cell cycle phase of U87 and U251 cells transfected with miR-NC or miR-346. **e** Western blot analysis of Cyclin E1, Cyclin D1 and CDK4 in U87 and U251 cells 48 h after transfection with miR-NC or miR-346. GAPDH served as a loading control. **P < 0.01, ***P < 0.001

To examine the functional roles of miR-346 in glioma, we used U87 and U251 cell lines for miR-346 overexpression. CCK8 was used to assay the proliferative capacity and showed that it was significantly decreased by lentivirus-mediated miR-346 overexpression (Fig. 2a). In addition, the colony numbers were lower compared with those in the control groups (Fig. 2b). Similarly, EDU assays indicated that DNA synthesis was suppressed in miR-346-overexpressing cells (Fig. 2c).

Changes in cell proliferation often reflect changes in cell cycle progression. Thus, we used flow cytometry to analyze the cell cycle. We found that miR-346 overexpression increased the percentage of cells in G0/G1 phase and decreased those in S and G2/M phases (Fig. 2d). Consistent with this finding, western blotting indicated that the cycle-related proteins Cyclin E1 and D1 were obviously reduced in the miR-346 overexpression group, while CDK4 remained unchanged (Fig. 2e). Taken together, these data suggest that miR-346 suppresses cell proliferation.

### NFIB is a direct target of miR-346 in glioma cells

**Fig. 3:**
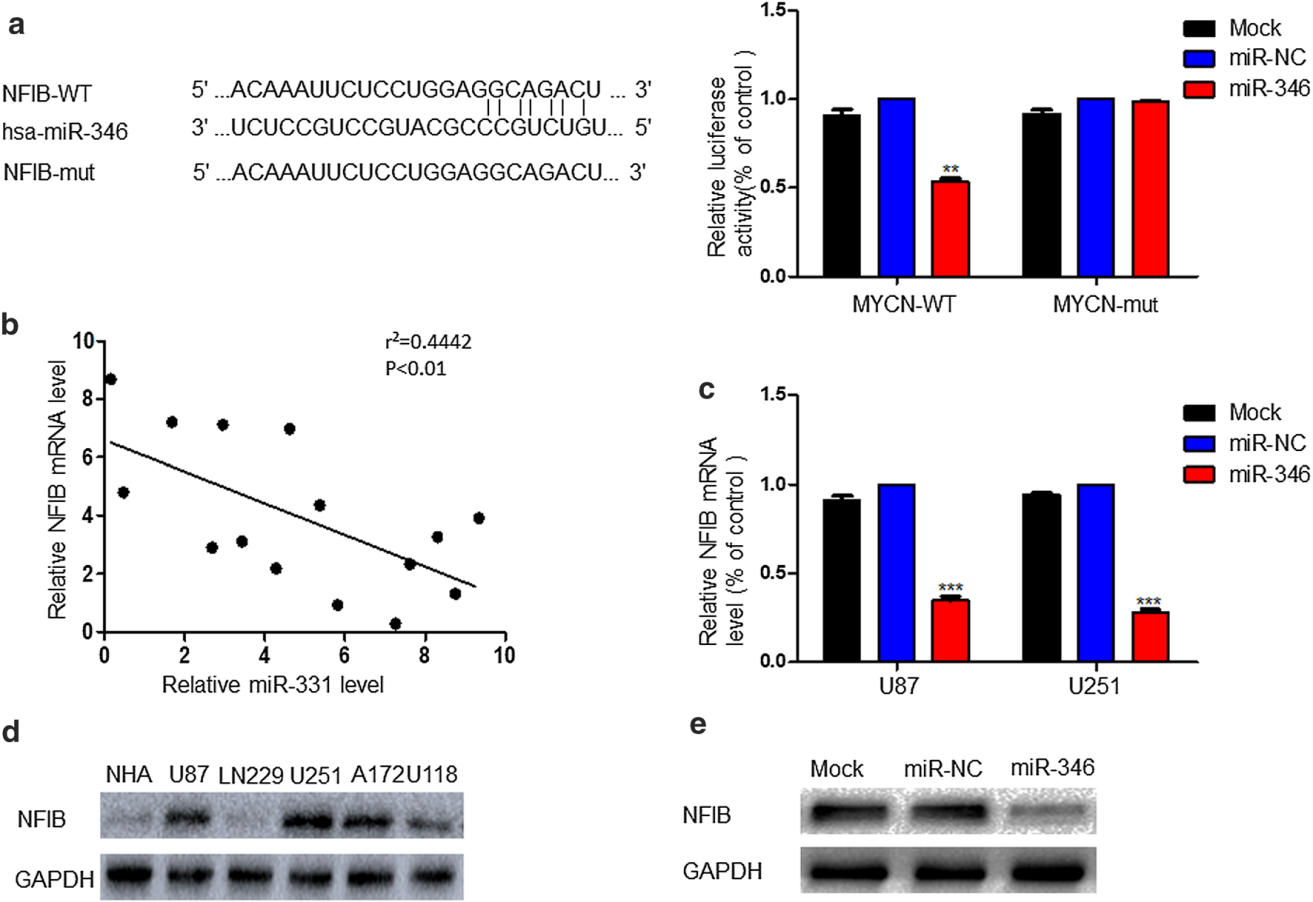
NFIB mRNA is a direct target of miR-346, and NFIB protein expression is inversely correlated with that of miR-346 in glioma tissues. **a** Left panel: predicted miR-346 target sequence in the wild-type (WT) 3′-UTR of NFIB mRNA and the mutated construct (mut). Right panel: Dual luciferase assay results. U87 and U251 cells were co-transfected with the recombinant luciferase plasmids. Luciferase activity of control group was normalized to 100%. **b** Spearman’s correlation analysis of NFIB protein and miR-346 expression levels in human glioma specimens (r^2^ = 0.4442, P < 0.01). **c** NFIB mRNA expression level in miR-346 over-expressed cells, detected by real-time PCR. GAPDH was used as internal control. **d** Western blot analysis of NFIB protein expression in normal human astrocytes (NHAs) and U87, LN229, U251, A172, and U118 glioma cell lines. **e** Western blot analysis of NFIB protein expression levels in U87 and U251glioma cells transfected with miR-NC or miR-346. **P < 0.01, ***P < 0.001

To understand the mechanism driving the influence of miR-346 on glioma cells, we accessed the commonly cited databases miRWalk 2.0 and TargetScan to identify potential miR-346 target genes. Consequently, we found NFIB, whose 3′ UTR contained sequence complementary to the seed sequence of miR-346. Next, we constructed a luciferase reporter vector and cotransfected it with miR-346 or miR-NC into U87 and U251 cells. The results showed that overexpression of miR-346 did not affect the luciferase activity of the NFIB 3′ UTR Mut reporter but decreased the luciferase activity of the WT reporter (Fig. 3a). Pearson’s correlation analysis showed that NFIB levels in GBM samples were inversely correlated with miR-346 levels (r^2^ = 0.4031, P = 0.0110) (Fig. 3b). In addition, overexpression of miR-346 reduced NFIB mRNA expression in the U87 and U251 cell lines (Fig. 3c). Western blotting confirmed that NFIB expression was obviously increased in U87 and U251 cells (Fig. 3d), and overexpression of miR-346 reduced NFIB expression (Fig. 3e).

### miR-346 inhibits the proliferation of glioma cells in an NFIB-dependent manner

**Fig. 4:**
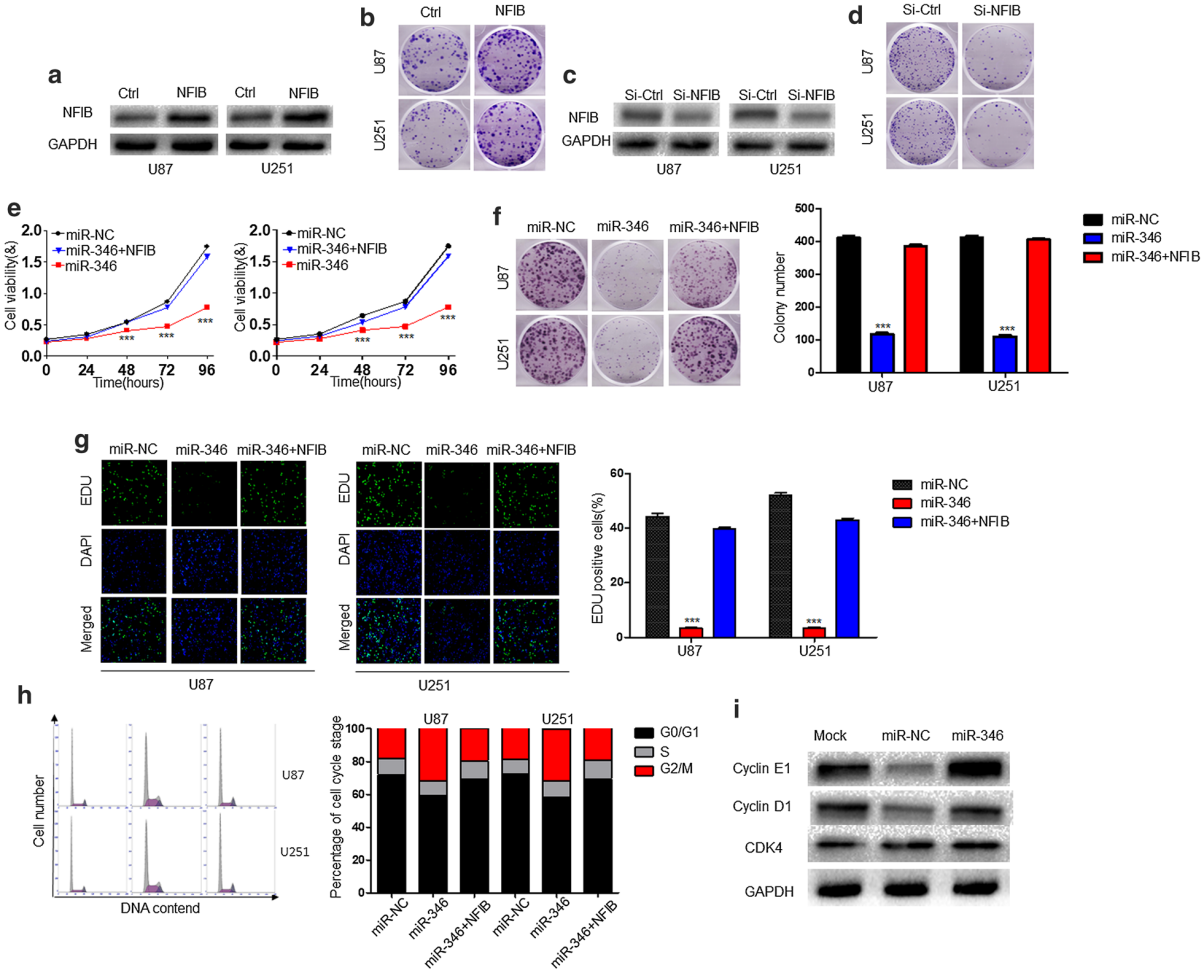
NFIB overexpression reverses the suppressive effects of miR-346 on glioma cells in vitro. **a** Western blot analysis of NFIB expression in U87 and U251 cells expressing control (Ctrl) or NFIB overexpression vectors. GAPDH served as the loading control. **b** Representative images and quantification of colony-forming assays of control or NFIB-overexpressing U87 and U251 cells. **c** Western blot analysis of NFIB expression in U87 and U251 cells after transfection with control (si-Ctrl) or NFIB-targeting siRNA (si-NFIB). GAPDH served as the loading control. **d** Representative images and quantification of colony-forming assays of U87 and U251 cells transfected with si-NFIB or si-Ctrl. **e**–**h** CCK-8 viability assay (**e**), colony-forming assay (**f**), EDU proliferation assay (**g**), and cell cycle distribution assay (**h**) of U87 and U251 cells transfected with miR-NC, miR-346, or miR-346 + NFIB. **i** Western blot analysis of NFIB and downstream effector proteins Cyclin E1, Cyclin D1 and CDK4 in U87 and U251 cells transfected with miR-NC, miR-346, or miR-346 + NFIB. GAPDH was used as the loading control. ***P < 0.001

As shown above, miR-346 and NFIB had a certain association. To confirm that NFIB was the downstream effector of miR-346, U87 and U251 cells were infected with control or NFIB expression lentivirus. Western blot analysis showed that NFIB expression was markedly increased in both cell lines (Fig. 4a), and overexpression of NFIB promoted colony-forming efficiency (Fig. 4b.) In contrast, knockdown of NFIB (Fig. 4c) suppressed the proliferation of U87 and U251 cells (Fig. 4d). These results suggest that miR-346 regulates the proliferation of glioma cells by directly targeting NFIB. Next, we validated whether overexpression of NFIB could reverse the effects of miR-346 overexpression. To this end, we cotransfected stable miR-346- or miR-NC-expressing U87 and U251 cells with a plasmid encoding human NFIB. As a result, overexpression of NFIB inhibited the miR-346-mediated suppression of cell proliferation, colony formation, and DNA synthesis (Fig. 4e–g). Moreover, NFIB expression alone reduced the number of cells in G0/G1 phase to inhibit cell proliferation (Fig. 4h). Furthermore, western blot analysis showed that Cyclin D1 and E1 protein levels were increased by NFIB expression, while CDK4 protein levels were unaffected (Fig. 4i).

### miR-346 overexpression inhibits tumor growth in vivo

**Fig. 5:**
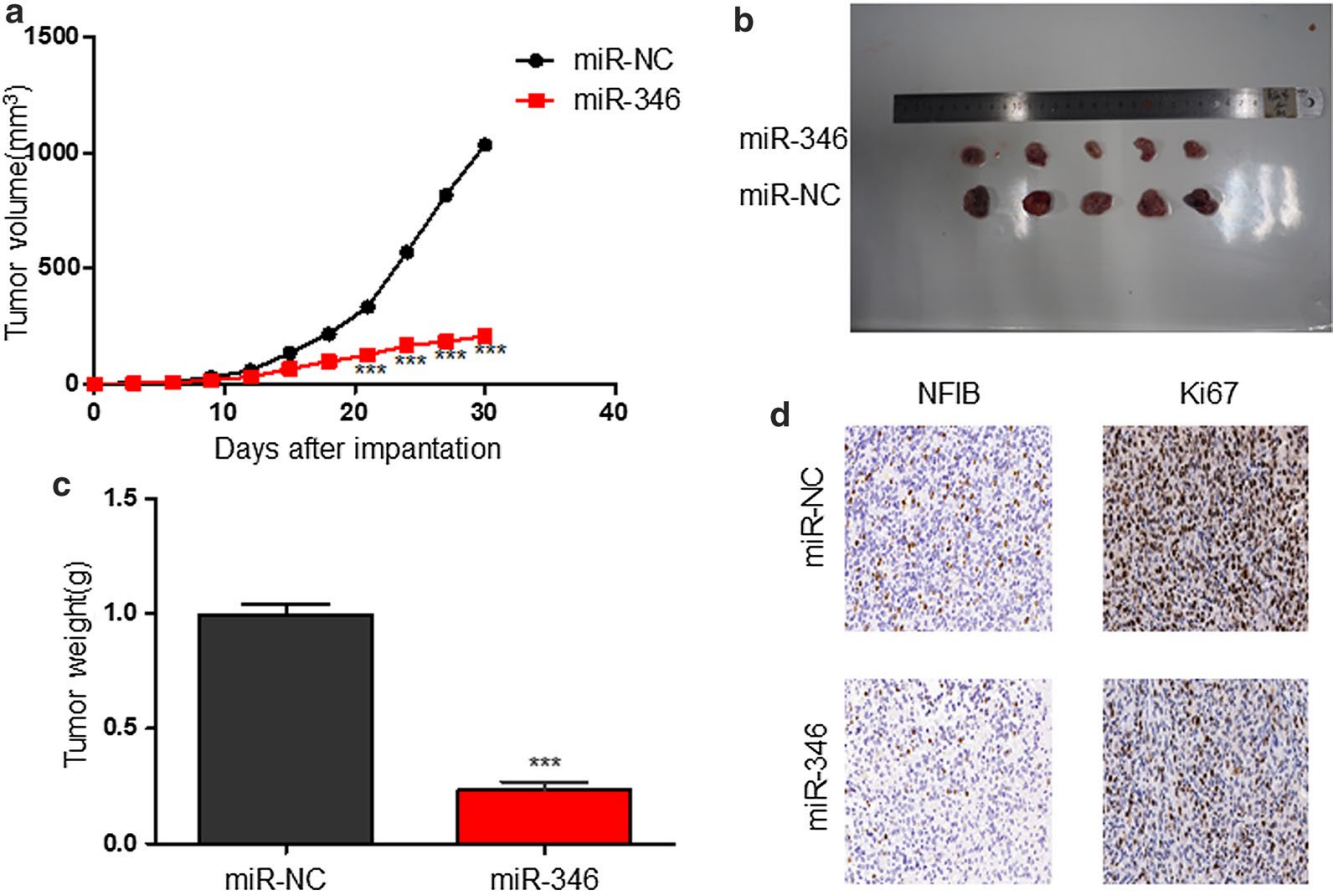
miR-346 overexpression suppresses glioma growth in vivo. **a** Tumor growth curves after subcutaneous injection of nude mice with U87 cells stably expressing miR-NC or miR-346 (n = 10). Tumor volumes were measured every 3 days from days 3 to 30. **b–d** Analysis of miR-NC- and miR-346-expressing U87 tumors on day 30 after injection: representative tumor images (**b**); tumor weights (**c**); immunohistochemical staining of NFIB and Ki-67 (**d**); ***P < 0.001

To validate the results of our functional experiments in vivo, we established tumor models in mice using human glioma cells. We generated lentiviral constructs to stably express miR-NC or miR-346 in U87 cells and then injected the cells into the flanks of athymic mice. Tumor sizes were measured every 3 days for 30 days at which point the mice were euthanized and their tumors were removed for analysis. Tumors with miR-346 overexpression were smaller (Fig. 5a, b) and weighed less (Fig. 5c) than those formed by miR-NC-transfected cells. IHC showed that expression of NFIB and proliferation marker Ki-67 was lower in miR-346-overexpressing tumors compared with control tumors (Fig. 5d). Collectively, these results suggest that miR-346 inhibits tumor growth in vivo by targeting NFIB.

## Discussion

GBM is reported to be the most common brain tumor; it has a poor prognosis and is difficult for clinicians to diagnose [19]. MicroRNAs (miRNAs) are small endogenous noncoding RNAs with lengths of approximately 18–23 nucleotides. Evidence suggests that miRNAs contribute to the progression of many kinds of tumors including GBM [20, 21]. miR-346 has been reported to be linked to many types of tumors, including hepatocellular carcinoma [20], renal carcinoma [22] and osteosarcoma [23]. In hepatocellular carcinoma, miR-346 promotes cell proliferation, migration and invasion by targeting KFL14 [20], and in renal carcinoma, miR-346 promotes cell growth by targeting GSK-3β [22]. In osteosarcoma, miR-346 inhibits tumor cell growth [23]. These results suggest that miR-346 is an important miRNA in tumors. However, the influence and molecular mechanisms of miR-346 in GBM are less studied.

In this study, through TCGA database analysis, we found that miR-346 expression was lower in GBM than in noncancerous brain tissues. To verify this, we subjected 15 glioma tissues and five noncancerous brain tissues to qRT-PCR and FISH analyses. The results showed that the expression of miR-346 was much lower in high-grade gliomas than in low-grade gliomas and particularly so for noncancerous brain tissues, indicating that miR-346 expression was strongly linked to glioma grade. Thus, miR-346 may be a new target for the treatment of glioma.

To investigate the influence exerted by miR-346 on glioma cells, we upregulated miR-346 expression in glioma cells by transfection of miR-346 using a lentivirus. Increased expression of miR-346 in glioma cells was closely associated with reduced proliferation and cell cycle arrest as shown by CCK8, colony formation, and EdU incorporation assays as well as flow cytometry. Western blotting showed that miR-346 inhibited the expression of the cycle-related proteins Cyclin E1 and D1 to affect cell cycle progression. We additionally predicted the targets of miR-346 using miRWalk2.0 and TargetScan. NFIB, a previously reported tumor promotor in many kinds of cancers [24], shared sequence complementarity in its 3′-UTR with the miR-346 seed sequence, which was confirmed by luciferase report assays. PCR and western blot results showed that overexpression of miR-346 downregulated the expression of NFIB, indicating a negative correlation. NFIB overexpression promoted the proliferation and colony-forming efficiency of glioma cells. Conversely, the effects of NFIB were inhibited by miR-346 overexpression, as shown by functional experiments. Therefore, miR-346 inhibited the proliferation of glioma cells by targeting NFIB in vitro, which was confirmed in vivo by animal experiments showing that overexpression of miR-346 inhibited tumorigenesis.

In summary, the present study provides convincing evidence to show a strong link between miR-346 and tumor growth. Our findings show that miR-346 functions as a tumor suppressor marker by negatively regulating NFIB expression. Nonetheless, further studies are needed to determine the precise mechanism of the interaction between miR-346 and NFIB and to explore more possible targets of miR-346. In addition, more experiments are needed to determine the function of NFIB in glioma.

## Conclusions

Our study provides strong evidence for a correlation between miR-346 and glioma. miR-346 suppressed the growth of glioma cells by directly targeting NFIB. Therefore, miR-346 may be a new biomarker and promising therapeutic target for glioma.

## Data Availability

All data generated or analyzed during this study are included in this published article.

## Abbreviations

miRNAs: microRNAs
TCGA: The Cancer Genome Atlas
miR-NC: miRNA negative control
siNC: si-non-coding
CCK8: Cell-Counting Kit 8
EDU: 5-ethynyl-2-deoxy-uridine
PBS: phosphate-buffered saline
SSC: saline sodium citrate
WT: wildtype
Mut: mutated
UTR: untranslated region
IHC: immunohistochemistry
